# Accuracy of HPV E6/E7 mRNA examination using in situ hybridization in diagnosing cervical intraepithelial lesions

**DOI:** 10.1186/s13000-021-01072-9

**Published:** 2021-02-19

**Authors:** Chang Hui, Huimin Bai, Jun Liu, Xuerong Lu, Shuzhen Wang, Zhenyu Zhang, Mulan Jin, Yue Wang, Yuzhen Liu

**Affiliations:** 1 Department of Obstetrics and Gynecology, Affiliated Hospital of Weifang Medical University, No. 2428, Yuhe Road, Weifang, 261042 China; 2 Department of Obstetrics and Gynecology, Beijing Chao-yang Hospital, Capital Medical University, No.8, North Road of Workers Stadium, Chaoyang District, Beijing, 100020 China; 3 Department of Pathology, Beijing Chao-yang Hospital, Capital Medical University, Beijing, China

**Keywords:** Cervical intraepithelial neoplasia, CIN, Diagnostic accuracy, E6/E7 mRNA, In situ hybridization

## Abstract

**Background:**

The consistency of pathologists in the diagnosis of cervical intraepithelial neoplasia (CINs) is not ideal, especially between low- and high-grade squamous intraepithelial lesions (LSIL and HSIL). This study was aimed to explore efficient strategies for the grading of CINs.

**Methods:**

The medical records of patients with high risk human papillomavirus (HR-HPV) infections who had underwent cervical biopsy or conization from April 2018 to April 2019 in Beijing Chao-Yang Hospital were collected and examined. The HR-HPV E6/E7 mRNA in the tissues of patients with CINs was detected using RNAscope chromogenic in situ hybridization (RISH). Immunohistochemistry (IHC) was performed to evaluate the expression of p16INK4a (P16) and Ki67.

**Results:**

HR-HPV E6/E7 mRNA signals were detected in 3/27 (11.1 %) of CIN 1, and in 32/33 (97.0 %) of CIN 2/3. Most of the staining patterns (27/32, 84.4 %) had a full-thickness epithelial layer staining with weak-to-strong nuclear and cytoplasmic dot-like signals in CIN 2/3, and there were also few special staining patterns that were significantly different from the others. A number of indicators were compared between LSIL and HSIL. There were statistically significant differences in E6/E7 mRNA, p16, Ki67 and cytology between the two groups (*P* < 0.05). According to the logistic regression analysis, merely E6/E7 mRNA positivity was significantly associated with CIN2/3 (OR: 52.53, 95 % CI, *P* < 0.05). In the detection of CIN 2/3, the sensitivity and specificity of HPV E6/E7 mRNA alone was not significantly inferior to that of its different combinations with Ki67, p16 and cytology (all, *P* > 0.05).

**Conclusions:**

RISH is efficient in grading of CINs. The HPV E6/E7 mRNA expression might reflect the phase HPV infections, and its positive pattern might predict the development direction of CINs, providing the possibility to realize more accurate treatments for patients.

## Background

Cervical cancer (CC) is one of the most common malignant tumors in women. Its incidence and mortality ranks fourth among malignant tumors in women, and approximately 300,000 women die due to this tumor every year [1]. A large number of studies have shown that HR-HPV persistent infection is the major tumorigenesis factor for CC and cervical intraepithelial neoplasia (CIN) [2]. CIN is the precursor lesion of CC, and is graded as 1, 2 and 3 [3]. The transformation from CIN to CC undergoes a gradual process, which requires an average of 5–14 years, when undetected or untreated [4]. Thus, early-diagnosis and timely-intervention are the key to reduce its incidence and mortality. The clinical management strategies for CINs have been based on the pathological diagnosis. However, the consistency of pathologists in the diagnosis of CINs remains unideal. Especially for CIN2, the reported diagnostic consistency of independent pathologists is less than 50 % [5, 6]. Due to the lack of reproducible histological distinctions and accurate biomarkers that could efficiently define the distinct intermediate state of CIN2, a two-tier system of low- and high-grade squamous intraepithelial lisions (LSIL and HSIL, respectively) has been recommended. Hence, CIN2 is no longer regarded as an independent histological subtype [3].

P16, as a multi-tumor suppressor gene, is directly involved in the regulation of the cell cycle, inhibiting the activity of cyclin-dependent protein kinase CDK4/CDK6 [7]. P16 overexpression has been detected in CINs and CC, and its level increases with the grade of the disease [8]. P16 has been used as a marker to improve the diagnostic consistency of CINs between different pathologists [9]. However, the natural history of p16, which present as positive in CIN1 and negative in CIN3 under certain circumstances, is still not well established [10, 11]. Thus, the downgrading or upgrading of diagnoses has been unavoidable, leading to under- or over-treatment for this patient group [4, 11].

The overexpression of HR-HPV E6/E7 genes is the key causative factor for CINs and CC [12]. The mRNA, as the direct transcription production of the gene, could accurately reflect its transcription activity. HR-HPV E6/E7 mRNA detection could effectively reduce the false positive rate of HPV DNA examinations due to the HPV transient infection. HPV E6/E7 mRNA (Aptima) detection with the technology of transcription mediated amplification (TMA) has been widely used in the screening of CC. However, this technology is not suitable for detecting samples of formalin-fixed paraffin-embedded (FFPE) tissues due to its low sensitivity, complicated operation, and other technical factors [13]. In situ hybridisation (ISH) is an established method for the detection and visualization of specific nucleic acid sequences (DNA and RNA) in tissue sections, cytological preparations, and whole organisms [14]. RNAscope technology is a new generation of single-molecule RNA ISH analysis technology. A novel probe design strategy and a hybridization-based signal amplification system to simultaneously amplify signals and suppress the background are applied during this procedure. The mRNA expression can be observed at the single-cell level under a standard bright-field microscope. This technology has the advantages of easy operation and high sensitivity [15]. RNAscope brings the benefits of in situ analysis to RNA biomarkers, and may enable the rapid development of RNA ISH-based molecular diagnostic assays. Several researchers have demonstrated that RNA ISH is more sensitive, when compared to DNA ISH, in terms of detecting HPV in oropharyngeal cancers [16, 17]. In the present analysis, RNA ISH was performed to detect the level of HR-HPV E6/E7 mRNA in the FFPE tissue of patients with CINs. The accuracy of this procedure in the grading of CINs was also evaluated.

## Methods

### Specimens

The medical records of patients with CINs detected by cervical biopsy or conization from April 2018 to April 2019 in Beijing Chao-Yang Hospital were retrospectively reviewed. All outpatients within 19–65 years old were routinely inquired whether they recently received CC screening examination. If not, cytology (HOLOGIC, USA) and HPV detection (Fluorometric Real-time PCR, Shanghai ZJ Bio-Tech, China) were performed under oral informed consent. The results for the cervical cytology were reported using the 2014 Bethesda (TBS) system [18]. According to the 2012 American Society for Colposcopy and Cervical Pathology (ASCCP) revised guidelines for the treatment of CC screening abnormal results [19], patients with abnormal cytology (worse than atypical squamous cells of undetermined significance, >ASC-US or continuous ASC-US), HR-HPV16/18 positive testing, persistent infection (more than one year) of the other subtypes of HR-HPV, ASC-US and HR-HPV positive testing, and/or visible suspicious lesions were scheduled for colposcopy. A biopsy was performed, when necessary. Most of the patients who were pathologically diagnosed with HSIL underwent physical therapy or loop electrosurgical excision procedure (LEEP). For young women with desire for fertility, patients with CIN2 were followed up when there was a satisfactory colposcopy which means that the cervix and squamocolumnar junction (SCJ) is fully visualized [19].

Patients with CIN1, CIN2 and CIN3 were compiled for further evaluation. Patients with concurrent invasive CC, which is a simultaneous or subsequent primary malignant tumor in other parts of the body, and incomplete medical records were excluded. Furthermore, patients with insufficient tissues for the ISH or IHC examination were also excluded.

The patient clinical data, which included age at diagnosis, smoking history, drinking history, age at first intercourse, sex partners, education, fertility status, menopausal status, body mass index (BMI), monthly income, cytology, HR-HPV detection, surgical method, pathological diagnosis and screening history, was collected and evaluated. The BMI was calculated by dividing the weight in kilograms by the height in meters squared. Subjects who smoked at least one cigarette a day and continuously for more than six months were considered positive for smoking history. Drinking at least once a month, including social engagements, was considered positive for drinking history.

The H&E slides of these patients were reviewed by two independent gynecologic pathologists, who were blind to the results. Any disagreements between these two reviewers were discussed and resolved. If a disagreement could not be resolved, a third review would be conducted until a consensus diagnosis (at least a two-way agreement) is reached. The histological classification criteria of CINs were referred to the World Health Organization (WHO) classification criteria for cervical intraepithelial lesions in 2014 [3]. For CIN1, the undifferentiated cells extended no more than one-third of the way up the epithelium. Nuclear hyperchromasia nuclear membrane irregularities and few mitotic features are usually present. Furthermore, koilocytosis can be observed. For CIN2, the undifferentiated cells were confined in the lower 2/3 of the epithelium, but exceeding the criteria for LSIL. Furthermore, these tended to more obviously present with nuclear atypia and mitotic features. For CIN3, differentiation and stratification may be totally absent, or only present in the superficial quarter of the epithelium. Nuclear abnormalities could be observed in the full-thickness epithelium. However, the lesion did not break through the basement membrane.

Five 4-µm-thick sections were cut from the FFPE tissue. Hematoxylin and eosin (H&E), RISH and IHC staining were respectively performed.

#### RNA RISH

The RNAscope HR 18 HPV assay is designed to detect the E6/E7 RNA for 18 HR HPV genotypes (HPV 16, 18, 26, 31, 33, 35, 39, 45, 51, 52, 53, 56, 58, 59, 66, 68, 73 and 82). The RNAscope® 2.5HD probes and detection kit were purchased from Advanced Cell Diagnostics (ACD, Hayward, USA). The assay was performed according to supplier’s instructions (ACD). Pretreatment: After being de-paraffinized and dehydrated, these sections were serially treated with the Pre-Treatment 1 solution and Pre-Treatment. Overnight, Pre-Treatment 3 was performed. Hybridization: The sections were hybridized in the HR 18 HPV hybridization solution without a cover slip in a HybEZ Oven (ACD, Hayward, USA). Signal amplification: The hybridized probe was performed through the serial application of Amp 1–6. Visualization: Diaminobenzidine (DAB) was used to demonstrate the amplified signal. The sections were counterstained with H&E, dehydrated with graded ethanol and xylene, and mounted with Cytoseal.

The staining data was recorded with the presence of dark brown, nuclear + and dot-like cytoplasmic+/−. The positivity pattern was recorded according to thickness of the epithelial staining, the presence and amount of diffuse, and/or the punctate nuclear staining and cytoplasmic staining, as well as the signal intensity. Morphologically, normal epithelia in the FFPE block were used as the internal negative control. A positive control was added to ensure that the dyeing process of the slides was successful. If the positive control did not show a positive staining, the batch of slides were considered as invalid.

The RISH slides were scanned using an automatic digital pathological slide scanner at 20 × magnification. The expression intensity of the HR-HPV E6/E7 mRNA was analyzed using the StrataQuest software. The nuclei were identified and screened by H&E staining channels. The effective nucleus, which was a single effective nucleus that eliminated the identified oversized adherent nuclei and undersized cell fragments, was used as the core to identify the areas with DAB staining signals within the cell range. Then, the E6/E7 mRNA expression of a single cell was quantified using the software, according to the DAB staining intensity and number of cells in the lesion area.

#### IHC was performed to detected the expression level of P16 and Ki67

IHC was performed using the antibodies of P16 (clone: G175-405, ZSGB-BIO, China) and Ki67 (clone: MIB1, ZSGB-BIO, China), respectively, according to manufacturer’s instructions. Then, the sections were de-paraffinized and dehydrated. The antigen retrieval was performed by boiling the slides with the ethylene diamine tetraacetic acid (EDTA) Antigen Retrieval solution (pH 9.0) in a pressure cooker for 15 min. After blocking the endogenous peroxidase with H_2_O_2_, the sections were incubated with the antibodies. Then, the secondary antibody reagent was performed. The reaction was detected by DAB and counterstained with H&E. A positive control slide was used to ensure the validity of the staining procedure.

Definition of P16 positivity: According to the Lower Anogenital Squamous Terminology (LAST) definition of p16 positivity [3], which calls “block positivity” continuous staining in the nucleus and/or cytoplasm, this was extended up from the basal layer through at least one-third of the epithelium. Definition of Ki67 positivity: Nuclear staining only and continuous staining, and this extended up from the basal layer through the lower third of the epithelium.

### Statistical analyses

SPSS (21.0) was used for the statistical analysis. The rate and percentage of expression was used to describe the general situation of the study subject. HSIL was used as the clinical endpoint of the disease to evaluate the diagnostic efficiencies of HPV E6/E7 mRNA ISH, P16 and P16/Ki67 on CINs, and calculate the sensitivity and specificity of these various detection methods. Chi-square was used to test for differences in expression rate, sensitivity and specificity. Binary logistic regression was used to calculate the OR and 95 % confidence interval (CI), which describes the risk factors associated with CINs. A *P*-value of < 0.05 was considered statistically significant.

## Results

A total of 67 patients, who underwent cervical biopsy or LEEP, were pathologically diagnosed with CINs during the study period. Five patients with concurrent invasive carcinoma were excluded due to invasive carcinoma. Two patients with insufficient tissue for ISH or IHC were excluded. Finally, a total of 60 patients, who met the inclusion criteria, were included for further evaluation. The clinicopathological characteristics of these patients are presented in Table 1. The mean age at diagnosis was 33.0 ± 8.8 years old (range: 18–65 years old). Furthermore, three women (5 %) were postmenopausal, 26 (43.3 %) women were nulliparous, and 34 (56.7 %) women were detected with abnormal cytology. In addition, positive p16 was detected in 36 (60 %) women, positive Ki67 was detected in 42 (70 %) women, and LSIL was detected in 27 (45 %) women. The remaining 33 (55 %) women were diagnosed with HSIL, which included CIN2 (17 cases) and CIN3 (16 cases), respectively. Positive HPV E6/E7 mRNA was identified in 35 (58.3 %) women, which included LSIL (three cases) and HSIL (32 cases), respectively. One patient with HSIL presented with negative HPV E6/E7 mRNA.

**Table 1:** Clinicopathological characteristics of the 60 patients with CINs

**Parameters**	**Numbers**	**Percent**
**Age at diagnosis, (Mean;range)**	33.0 ± 8.8	18–65
**Smoking history**		
**Yes**	5	8.3%
**No**	55	91.7%
**Drinking**		
**Yes**	10	16.7%
**No**	50	83.3%
**Education**		
**Primary**	1	1.7%
**Secondary**	20	38.3%
**College and above**	36	60.0%
**Fertility status**		
**Nulliparous**	26	43.3%
**Pluriparous**	34	56.7%
**Menopausal status**		
**Pre-menopausal**	57	95%
**Post-menopausal**	3	5%
**BMI**		
**Normal(18.5-25)**	46	76.7%
**Overweight(> 25)**	6	10.0%
**Underweight(< 18.5)**	8	13.3%
**Monthly income**		
**≤10000**	34	56.7%
**>10000**	26	43.3%
**Presentation**		
**Contact bleeding**	3	5%
**Symptomless**	57	95%
**Screening history**		
**Ever**	27	45%
**Never**	33	55%
**Age at first intercourse (Mean;range)**	22.0 ± 2.6	18-31
**Sex partners**		
**One**	42	70.0%
**More than one**	18	30.0%
**Referral Cytology**		
**NILM**	26	43.3%
**ASC-US**	12	20.0%
**ASC-H**	5	8.3%
**LISL**	9	15.0%
**HISL**	8	13.3%
**HR-HPV infection**		
**Yes**	60	100%
**No**	0	
**CINs detected by**		
**Biopsy**	16	26.7%
**LEEP/CKC**	44	73.3%
**Pathological diagnosis**		
**CIN1**	27	45.0%
**CIN2**	17	28.3%
**CIN3**	16	26.7%
**E6/E7mRNA**		
**Positive**	35	58.3%
**Negative**	25	42.7%
**p16**		
**Positive**	36	60%
**Negative**	24	40%
**Ki67**		
**Positive**	42	70%
**Negative**	18	30%

Monthly income was the average monthly income of an individual family

*NILM* Negative for intraepithelial lesion or malignancy, *ASCUS* Atypical squamous cells of undetermined significance, *ASC-H* Atypical cells cannot exclude high grade, *BMI* Body mass index

**Fig. 1:**
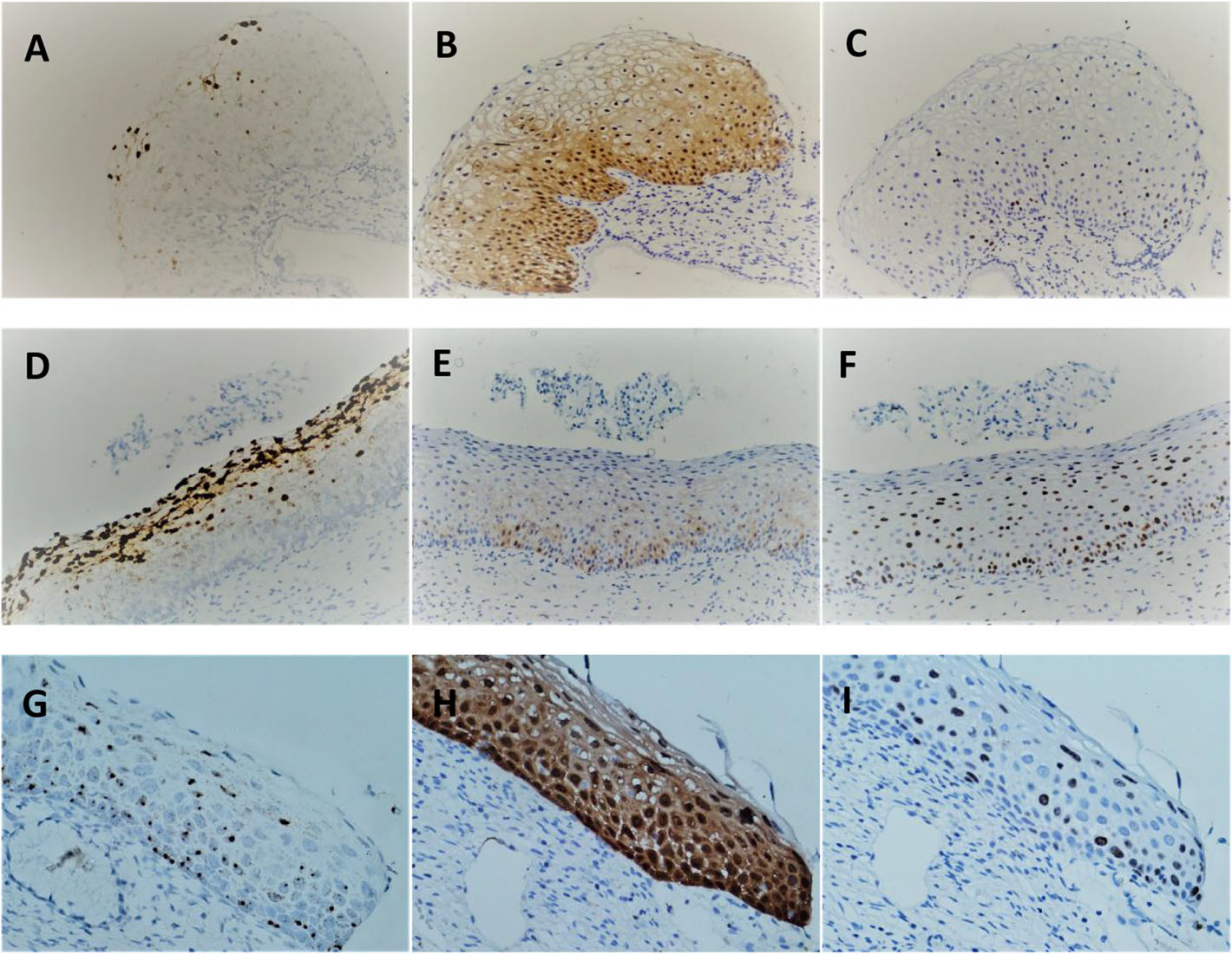
CIN staining results. The CIN1 specimen (**a**-**c**), **a** HPV E6/E7 mRNA positive, **b** p16 positive, **c** Ki67 positive. CIN2 specimen (**d**-**f**), **d** HPV E6/E7 mRNA positive, **e** p16 negative, the signals were limited in the basal layer. **f** Ki67 positive. CIN3 specimen (**g**-**i**), **h** HPV E6/E7 mRNA positive, **i** p16 positive, **g** Ki67 positive

**Fig. 2:**
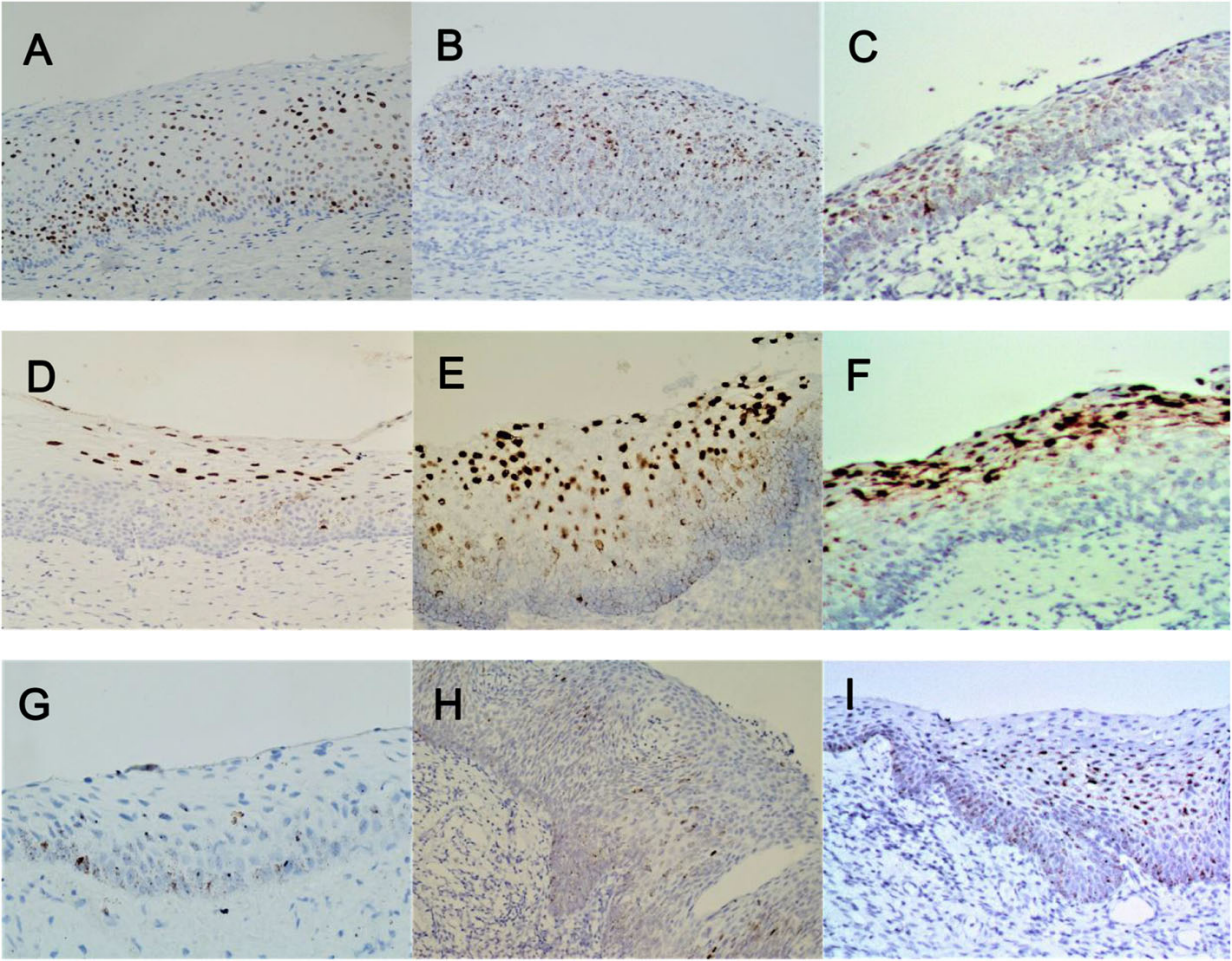
HPV E6/E7 mRNA staining patterns. According to the locations of the signals: BSE (**a**-**c**): basal up to superficial epithelium, UME (**d**-**f**): signal staining confined in upper middle epithelial layer. LME (**g**-**i**): signal staining confined in the lower middle epithelial layer

The characteristics of the HPV E6/E7 mRNA staining of patients presented with an E6/E7 mRNA positive expression and LSIL disease, as presented in Fig. 1. The nucleus and cytoplasm in the full-thickness epithelial layer presented with dot-like signals, and different epithelial layers presented with diffuse nuclear signals. For most of the (27/33, 81.8 %) patients with HSIL disease, the positive staining of HPV E6/E7 mRNA presented with weak-to-strong nuclear and cytoplasmic dot-like signals within full-thickness epithelial layer (Fig. 2). In addition, the positive patterns of CIN2 and CIN3 were respectively observed, According to the locations of the signals, the following expression was used for recording (Table 2): For CIN2, signals extending to the basal, up to the surface epithelium, were observed in 62.5 % of the patients; signals confined in the upper middle epithelial layers were observed in 31.3 % of the patients; signal staining confined in the lower middle epithelial layers was observed in 6.2 % of the patients. For CIN3, a case of indeterminate depth of expression was excluded due to inadequate epithelium. Signals extending to the basal, up to the surface epithelium, were observed in 66.7 % of the patients; signals confined in the upper middle epithelial layers were observed in 20.0 % of the patients; signal staining confined in the lower middle epithelial layers were observed in 13.3 % of the patients. Although there were some differences between these, there was no statistical significance (*P* > 0.05). The analysis of the StrataQuest digital images revealed that there was no statistical difference in single-cell intensity or the percentage of positive cells between CIN2 and CIN3 (Table 3, *P* > 0.05).

**Table 2:** Staining patterns of HPV E6/E7 mRNA in situ hybridization in CIN2 and CIN3 n(%)

	**CIN2+ (*n* = 16)**	**CIN3+ (*n* = 15)**
**BSE**	10 (62.5)	10 (66.7)
**UME**	5 (31.3)	3 (20.0)
**LME**	1 (6.2)	2 (13.3)

*UME* Signals staining confined in upper middle epithelial layers, *LME* Signals staining confined in lower middle epithelial layers, *BSE* Signals staining basal up to surface epithelium

**Table 3:** Comparison between CIN2 and CIN3 in images analysis

	Grouping		*t*	*P*
	CIN2 (*n* = 16)	CIN3 (*n* = 15)		
Percentage of positive cells	44.68 ± 26.31	43.55 ± 22.73	0.126	0.901
Single-cell Intensity	27.50 ± 14.55	19.73 ± 6.69	1.930	0.067

The potential predictors for HSIL disease were evaluated in Tables 4 and 5. In the univariate analysis, positive E6/E7 mRNA, p16, Ki67 and abnormal cytology (≥ ASC-US) were statistical risk factors for HSIL (*P* = 0.000, 0.000, 0.000 and 0.024, respectively). Positive E6/E7mRNA was a risk factor for HSIL in the logistic regression analysis (*P* = 0.011), with an OR of 3.961, indicating that HSIL Positive RNA was 3.961 times more likely than negative RNA. For patients with positive E6/E7 mRNA expression, the risk rate of HSIL reached as high as 91.4 %, when compared to the rate of 4.0 % for those with negative E6/E7 mRNA expression.

**Table 4:** The distribution of indicators characteristics and risk factors

**The baseline data**	**Grouping**		*P*
	LSIL (*n* = 27)	HISL (*n* = 33)	
**Age**			
≤ 33	16	17	0.55
> 33	11	16	
**Fertility status**			
No	25	32	0.50
Yes	2	1	
**Cytology**			
Normal	16	10	0.04
Abnormal	11	21	
**Menopausal status**			
No	13	13	0.44
Yes	14	20	
**Age at first intercourse**			
≤22	13	20	0.34
>22	14	13	
**Sex partners**			
1	21	21	0.23
> 1	6	12	
**Education**			
Bachelor below	13	11	0.05
Bachelor or above	14	22	
**Monthly income**			
≤1w	17	17	0.37
>1w	10	16	
**Smoking history**			
-	24	1	0.24
+	3	32	
**Drinking**			
-	22	2	0.73
+	5	31	
**Screening history**			
Ever	15	18	0.94
Never	12	15	
**BMI**			
18.5-25	22	24	0.72
>25	2	4	
<18.5	3	5	
**E6/E7mRNA**			
-	24	1	0.00
+	3	32	
**p16**			
-	22	2	0.00
+	5	31	
**Ki67**			
-	17	1	0.00
+	10	32

**Table 5:** Logistic regression model to estimate the association between the risk factors and CIN2/3

	B	*P*	OR(95% C.I.)
**E6/E7mRNA**	3.961	0.011	52.53
**p16**	20.452	0.998	7.62E8
**Ki67**	1.099	0.636	3.00
**Cytology**	0.777	0.602	2.18

The sensitivity and specificity for the HPV E6/E7 mRNA was 96.97 % and 88.89 %, respectively (Table 6). The sensitivity and specificity for the HPV E6/E7 mRNA alone were statistically better, when compared to p16 and p16/Ki67. However, these were not significantly inferior to the different combinations (all, *P* > 0.05).

**Table 6:** Clinical performance of various indicators for detection of CIN2/3

	**Pathological diagnosis**		**Sensitivity**	***P***	**Specificity**	***P***
	LSIL (*n* = 27)	HISL (*n* = 33)				
**E6/E7mRNA**						
+	3	32	96.97%	0.555	88.89%	0.444
-	24	1				
**p16**						
+	5	31	93.94%	0.555	81.48%	0.444
-	22	2				
**p16/Ki67**						
+	4	30	90.91%	0.302	85.19%	0.685
-	23	3				
**E6/E7mRNA/p16**						
+	2	30	90.91%	0.302	92.59%	0.639
-	25	3				
**E6/E7mRNA/Ki67**						
+	3	31	93.94%	0.555	88.89%	1.000
-	24	2				
**E6/E7mRNA/p16/Ki67**						
+	2	29	87.88%	0.163	92.59%	0.639
-	25	4				

## Discussion

The present study had three major findings: (1) HPV E6/E7 mRNA was highly expressed in HSIL, and its positive expression obviously increased with the grade of the lesion. (2) For the detection of HSIL, the sensitivity and specificity of HPV E6/E7 mRNA ISH were better than p16 and p16/Ki67, but were not significantly inferior to the other different combinations. (3) The HPV E6/E7mRNA positive expression might reflect the phase of HPV infection, and its expression pattern may predict the development direction of CINs.

In the present study, HPV E6/E7 mRNA, Ki67, p16 and cytology were the predictors of HSIL in the univariate analysis. Cytology examination (ThinPrep cytology test, TCT) has been widely used for the screening of CC. A large prospective multicenter clinical study revealed that the sensitivity and specificity of TCT is 73 %-94 % and 58 %-76 %, respectively, in the screening of CC. Compared with the traditional pap smear, the detection of TCT for LSIL and above could reach as high as 73 % [20]. However, this technology is not suitable for the detection samples of FFPE tissues due to its technology [21]. P16 and Ki67 were the most widely used biomarkers for the grading of CINs [22]. Studies have shown that the combination of H&E and p16 or Ki67 is a good approach to improve the accuracy of the pathological diagnosis of CINs [22, 23]. However, studies have argued that the overexpression of P16 may be closely correlated to the HPV oncoprotein E7 inactivating cell-cycle regulation Rb protein [24]. At present, the specific mechanism is not fully understood, and the correlation between P16 and history of HPV infection remains unclear. P16 expression status cannot accurately predict the potential risk of CINs. Furthermore, the negative expression of p16 does not exclude HPV infection, and p16 positive does not mean HPV infection or HSIL results [25, 26]. This is consistent with the present data. In the present study, the sensitivity and specificity of p16 to the detection of HSIL was 93.94 % and 81.48 %, respectively. In addition, a previous study revealed that p16 is not only expressed in high grade lesions and Caski cervical cancer cells (ATCC® CRM-CRL-1550), but also in normal cervical cells, and the single-cell intensity between CIN cells and Caski cells were similar [27]. Although this result may be due to the reason that the cervix cells from the original generation of culture had the capacity of proliferation, these cells were HPV negative. Hence, the correlation between p16 and HPV infection needs to be further studied. Some studies also reported that the interpretation of p16 was subjective, and that there are no criteria widely accepted for what the exact cut-off point for a positive vs. negative immunohistochemical stain should be [3, 28].

As a nuclear proliferation antigen, ki67 is exclusively expressed in the proliferation phase of the cell cycle [29]. Ki67 expression in normal cervical tissues, CIN and cervical cancer tissues gradually increases with the aggravation of the disease. Furthermore, there is a statistical difference in the positive expression between LSIL and HSIL [30]. P16 overexpression occurs only when the cell cycle modulation is disordered, and the cell proliferation becomes abnormal. Under normal physiological conditions, Ki67 and p16 are not simultaneously expressed in the same cell. The co-expression of Ki67 and p16 usually indicates that the cell cycle is out of control, and that abnormal cell proliferation occurs [31]. The use of Ki67 or p16/Ki67 significantly improved the accuracy of the pathological diagnosis of cervical lesions [32]. However, ki67 was overexpressed in inflammation, LSIL, and a variety of benign tumors [33]. ki67 negative expression cannot exclude the existence of HPV infection and CINs, especially LSIL. Studies have also reported that Ki-67 and p16/Ki67 exhibited no marked improvement over p16 alone in the grading of CINs [3, 28]. Hence, the routine addition of Ki-67 to p16 is not recommended, which was consistent with the results of these present studies. In the present study, the sensitivity and specificity of p16 was 93.94 % and 81.48 % in the detection of HSIL. p16/Ki67 detection was 90.90 % and 85.19 %, which was not significantly inferior to p16 alone.

In the early stage of HPV infection, the virus mostly presents as a transient infection or latent infection. The HPV genome is in a free state in the nucleus, and maintains a low replication activity [34]. HPV E6/E7 genes are regulated by E1/E2 genes, and their expression is inhibited during this stage. E6/E7 mRNA is often undetectable. This phase often occurs in normal cervical tissues or CIN1 [24, 35]. In the present analysis, the negative expression of E6/E7 mRNA was identified in 88.9 % of patients with LSIL. In LSIL, the images of the HPV E6/E7 mRNA staining in the nucleus and cytoplasm in the full-thickness epithelial layer presented with dot-like signals, and the different epithelial layers presented with diffuse nuclear signals. During the persistent infection stage, the HR-HPV DNA integrated into the host genome with the disruption and loss of the E2 gene, and this lead to the HPV E6/E7 mRNA overexpression. This phase mostly occurs in CIN2/3 [24, 35]. In the present study, 97.0 % of patients with HSIL were detected with a positive expression of HPV E6/E7 mRNA. The typical HPV E6/E7 mRNA image of HSIL presented with weak-to-strong nuclear and cytoplasmic dot-like signals within the full-thickness epithelial layer. HPV E6/E7 mRNA was detected in almost CIN2/3, which exhibited a transition phase. Evans et al. [36] investigated the application of HPV E6/E7 mRNA in situ hybridization in the three-tier grading of CINs. They detected the HR-HPV E6/E7 mRNA in 86 cases with CINs lesions, and confirmed that its expression in HSIL was 100 %. The assay supports that RISH can be used in CIN histological grading, and that the HPV E6/E7 mRNA expression reflect the phase of the HPV infection. In our study, we added Ki67 and P16/ Ki67 as the control group. The positive E6/E7 mRNA was identified as the only independent risk factor for HSIL(*P* = 0.011,OR: 3.961) in the logistic regression analysis. The sensitivity and specificity of HPV E6/E7 mRNA alone were better than p16, abnormal cytology and Ki67, and were not significantly inferior to the different combinations. E6/E7 mRNA was the direct transcription production of the gene. The expression level of E6/E7 mRNA could accurately reflect the status of the HPV infection. Therefore, it was concluded that RISH can be used for the grading of cervical lesions, and that this has the potential to reflect the different stages of HPV infection.

The progressing risk of HSIL was significantly higher than that of LSIL. Furthermore, approximately a third of the patients could naturally regress, especially for CIN2 in young women, and this natural regression increased with the extension of time [37]. Most young women are eager to have children, and unnecessary clinical intervention may impair cervical function, lead to infertility or other cervix related problems, and impose an unnecessary financial burden [38]. For CIN2, HPV E6/E7 mRNA were detected in 94.1 % of patients. Furthermore, signals extending to the basal, up to the surface epithelium, were observed in 62.5 % of the patients, and signals confined in the upper middle epithelial layers were observed in 31.3 % of the patients. For CIN3, a case of indeterminate depth of expression was excluded due to inadequate epithelium. HPV E6/E7 mRNA were detected in 100 % of the patients. Furthermore, signals extending to the basal, up to the surface epithelium, were observed in 66.7 % of the patients, and signals confined in the upper middle epithelial layers were observed in 20.0 % of the patients. Although there were some differences between these, there was no statistical significance. The intensity of single cells and the percentage of positive cells between CIN2 and CIN3 were compared using the StrataQuest digital image analysis software. The results revealed that there was no statistical significance. These data support the 2-tiered system with LSIL and HSIL. According to the understanding of HPV biology, the biological behavior between CIN2 and CIN3 is indistinguishable [3, 39]. In the 2-tiered system, LSIL reflected the transient HPV infection, while HSIL reflected the persistent HPV infection, which has the potential to progress to CC. In addition, it was observed in two special samples that the E6/E7 mRNA positive patterns were limited in the basal layer with a weak signal in HSIL, which was significantly different from the others. Studies have shown that HPV E6/E7 mRNA is usually highly expressed in HSIL [20, 34]. However, the HPV E6/E7mRNA of the two samples were expressed at a low level, which reflects that the lesion might be in the phase of regression. At the same time, one sample of HSIL presented with negative expression. It was speculated that the reasons might be, as follows: (1) the lesion was in the phase of regression; (2) the possibility of mRNA degradation in tissue sections; (3) the HPV test may be false positive. HPV E6/E7mRNA directly reflects the state of HPV transcription. Hence, it was speculated that the RISH positive pattern might predict the development direction of CINs, which is worthy of further investigation.

Although the present study has some limitations, which included the relative small sample size and insufficient follow-up data, the present data provides an objective and convincing method to assist in the grading of cervical lesions.

In summary, the data of the present study support the view that HPV E6/E7 mRNA ISH can be used to assist in the grading of CINs. HPV E6/E7 mRNA positive expression might reflect the phase of the HPV infection, and its positive pattern may predict the development direction of CINs, which could provide the possibility to realize a more accurate treatment approach for patients. There is a huge potential value in pathological diagnosis of CINs with HPV E6/E7 mRNA ISH, which is worthy of further investigation.

## Data Availability

The datasets used and/or analysed during the current study are available from the corresponding author on reasonable request.

## Abbreviations

ACD: Advanced Cell Diagnostics
ASC-H: Atypical cells cannot exclude high grade
ASC-US: Atypical squamous cells of undetermined significance
ASCCP: American Society for Colposcopy and Cervical Pathology
BMI: Body mass index
CIN: Cervical intraepithelial neoplasias
CKC: Cold knife conigation
CAP: The College of American Pathologists
DAB: Double Amino benzidine
FFPE: Formalin-fixed paraffin-embedded
HE: Hematoxylin-eosinstaining
HSIL: High-grade squamous intraepithelial lesion
HPV: Human papilloma virus
HR-HPV: High risk-human papilloma virus
HC-2: Hybrid capture-2
ISH: In situ hybridization
LAST: Lower Anogenital Squamous Terminology
LSIL: Low-grade squamous intraepithelial lesion
LEEP: Loop electrosurgical excision
NILM: Negative for intraepithelial lesion or malignancy
RISH: RNAscope chromogenic in situ hybridization
SCJ: Squamocolumnar junction
TMA: Transcription mediated amplification
